# Time to Functional Recovery After Laser Tonsillotomy Performed Under Local Anesthesia vs Conventional Tonsillectomy With General Anesthesia Among Adults: A Randomized Clinical Trial

**DOI:** 10.1001/jamanetworkopen.2021.48655

**Published:** 2022-02-21

**Authors:** Justin E. R. E. Wong Chung, Rozemarie van Geet, Noud van Helmond, Chloé Kastoer, Stefan Böhringer, Wilbert B. van den Hout, Hendrik P. Verschuur, Ferdinand A. W. Peek, Patrick F. M. Dammeijer, Gijs K. A. van Wermeskerken, Peter Paul G. van Benthem, Henk M. Blom

**Affiliations:** 1 Department of Otolaryngology Head and Neck Surgery, Hagaziekenhuis, The Hague, the Netherlands; 2 Department of Otolaryngology Head and Neck Surgery, Leiden University Medical Center, Leiden, the Netherlands; 3 Department of Otolaryngology Head and Neck Surgery, Antwerp University Hospital, Antwerp, Belgium; 4 Department of Anesthesiology, Cooper University Medical School of Rowan University, Cooper University Health Care, Camden, New Jersey; 5 Department of Otolaryngology Head and Neck Surgery, Haaglanden Medical Center, The Hague, the Netherlands; 6 Department of Medical Statistics, Leiden University Medical Center, Leiden, the Netherlands; 7 Department of Biomedical Data Sciences, Leiden University Medical Center, Leiden, the Netherlands; 8 Department of Otolaryngology Head and Neck Surgery, Reinier de Graaf, Delft, the Netherlands; 9 Department of Otolaryngology Head and Neck Surgery, VieCuri Medical Center, Venlo, the Netherlands; 10 Department of Otolaryngology Head and Neck Surgery, Amphia Hospital, Breda, the Netherlands

## Abstract

**Importance:**

Carbon dioxide laser tonsillotomy performed under local anesthesia may be an effective and less invasive alternative than dissection tonsillectomy for treatment of tonsil-related afflictions.

**Objective:**

To compare functional recovery and symptom relief among adults undergoing tonsillectomy or tonsillotomy.

**Design, Setting, and Participants:**

This randomized clinical trial was conducted at 5 secondary and tertiary hospitals in the Netherlands from January 2018 to December 2019. Participants were 199 adult patients with an indication for surgical tonsil removal randomly assigned to either the tonsillectomy or tonsillotomy group.

**Interventions:**

For tonsillotomy, the crypts of the palatine tonsil were evaporated using a carbon dioxide laser under local anesthesia, whereas tonsillectomy consisted of total tonsil removal performed under general anesthesia.

**Main Outcomes and Measures:**

The primary outcome was time to functional recovery measured within 2 weeks after surgery assessed for a modified intention-to-treat population. Secondary outcomes were time to return to work after surgery, resolution of primary symptoms, severity of remaining symptoms, surgical complications, postoperative pain and analgesics use, and overall patient satisfaction assessed for the intention-to-treat population.

**Results:**

Of 199 patients (139 [70%] female; mean [SD] age, 29 [9] years), 98 were randomly assigned to tonsillotomy and 101 were randomly assigned to tonsillectomy. Recovery within 2 weeks after surgery was significantly shorter after tonsillotomy than after tonsillectomy (hazard ratio for recovery after tonsillectomy vs tonsillotomy, 0.3; 95% CI, 0.2-0.5). Two weeks after surgery, 72 (77%) patients in the tonsillotomy group were fully recovered compared with 26 (57%) patients in the tonsillectomy group. Time until return to work within 2 weeks was also shorter after tonsillotomy (median [IQR], 4.5 [3.0-7.0] days vs 12.0 [9.0-14.0] days; hazard ratio for return after tonsillectomy vs tonsillotomy, 0.3; 95% CI, 0.2-0.4.). Postoperative hemorrhage occurred in 2 patients (2%) in the tonsillotomy group and 8 patients (12%) in the tonsillectomy group. At 6 months after surgery, fewer patients in the tonsillectomy group (25; 35%) than in the tonsillotomy group (54; 57%) experienced persistent symptoms (difference of 22%; 95% CI, 7%-37%). Most patients with persistent symptoms in both the tonsillotomy (32 of 54; 59%) and tonsillectomy (16 of 25; 64%) groups reported mild symptoms 6 months after surgery.

**Conclusions and Relevance:**

This randomized clinical trial found that compared with tonsillectomy performed under general anesthesia, laser tonsillotomy performed under local anesthesia had a significantly shorter and less painful recovery period. A higher percentage of patients had persistent symptoms after tonsillotomy, although the intensity of these symptoms was lower than before surgery. These results suggest that laser tonsillotomy performed under local anesthesia may be a feasible alternative to conventional tonsillectomy in this population.

**Trial Registration:**

Netherlands Trial Register Identifier: NL6866 (NTR7044)

## Key Points

**Question:**

Does laser tonsillotomy performed under local anesthesia offer comparable symptom relief but shorter functional recovery times than conventional tonsillectomy performed under general anesthesia among adults undergoing surgical tonsil removal?

**Findings:**

This randomized clinical trial of 199 adults found that compared with tonsillectomy, functional recovery was quicker after laser tonsillotomy although resolution of the chief concern leading to surgery was lower.

**Meaning:**

Depending on individual patient preferences, laser tonsillotomy performed under local anesthesia may be a feasible alternative to conventional tonsillectomy performed under general anesthesia among adults.

## Teaser

This randomized clinical trial compares functional recovery times and persistence of chief symptoms after conventional tonsillectomy performed under general anesthesia with laser tonsillotomy performed under local anesthesia among adults undergoing surgical tonsil removal for treatment of tonsil-related afflictions.

## Introduction

Tonsillitis, peritonsillar abscess, tonsillolithiasis, halitosis, dysphagia, and snoring are common tonsil-related conditions in adults. When conservative treatment of these conditions fails, surgery may be indicated.^1^

Classic dissection tonsillectomy with complete tonsil removal under general anesthesia is the most used surgical technique. In the United States and Europe combined, more than 500 000 tonsillectomies are performed in adult patients every year.^2,3^ After tonsillectomy, recovery to normal function is typically long. Postoperative complications of tonsillectomy include bleeding, infection, and severe pain, any of which may lead to hospital readmissions and contribute to a protracted recovery.^4^ Partial removal of the tonsil, tonsillotomy, has been performed for 3000 years and is increasingly being reexplored to potentially decrease patient burden and risk.^5^ During tonsillotomy, only the cryptic lymphatic tissue is removed, and the tonsil capsule that contains larger nerves and blood vessels is left intact.^6,7^ This may lead to less postoperative pain and bleeding.^7,8,9^ Tonsillotomy may be performed in adults using different instruments and techniques, including the use of carbon dioxide (CO_2_) laser, diathermy, radiofrequency, microdebrider, coblation, bipolar electrosurgical device, and cold steel dissection.^6,10^

The CO_2_ laser is the most used laser modality in tonsillotomy and is known for its good ablation and hemorrhage-controlling characteristics.^11,12^ An advantage of the CO_2_ laser is its ability to perform tonsillotomy under local anesthesia without sedation.^6^ General anesthesia has additional effects on postoperative and functional recovery, and obviating the need for general anesthesia may provide additional recovery benefit over tonsillectomy.^13^ However, there is a lack of evidence with sufficient quality regarding the clinical usefulness of tonsillotomy compared with tonsillectomy in adults.^5^

We conducted a randomized clinical trial to compare CO_2_ laser tonsillotomy performed under local anesthesia to tonsillectomy performed under general anesthesia for tonsil-related concerns in adults. We hypothesized that tonsillotomy would have a shorter functional recovery period. Secondary outcomes included relief 6 months after surgery of the primary concern that led to tonsil removal and tonsil symptom severity.

## Methods

The laser tonsillotomy vs tonsillectomy study (TOMTOM study) is a pragmatic randomized clinical trial (meaning that broad inclusion criteria comparable to a real-world situation were applied) comparing functional recovery time, resolution or reduction of tonsil concerns, and surgical complications between tonsillotomy and tonsillectomy in adults. Patients were recruited from 5 otolaryngology departments of teaching hospitals in the Netherlands from January 2018 to December 2019. This report followed the Consolidated Standards of Reporting Trials (CONSORT) reporting guideline.^14^ The study protocol and the statistical analysis plan are available in Supplement 1. The study was approved by the Research Ethics Committee of The Hague in the Netherlands. This study adhered to Dutch health care laws and the principles set forth in the Declaration of Helsinki.^15^ All patients provided written informed consent. No one received compensation or was offered any incentive for participating in this study.

### Patients

Adult patients (age ≥18 years) with tonsil-related concerns that did not resolve sufficiently with conservative management, including antibiotic treatment, were eligible for enrollment in the study. Patients with the following conditions, similar to real-life standard of care practice, were included: chronic or recurrent tonsillitis (indication for surgery was determined following the Dutch guideline of >4 tonsillitis episodes per year not responding sufficiently to antibiotic treatment^16^), tonsil-related halitosis, tonsillolithiasis, dysphagia, and sleep apnea caused by the tonsils. Exclusion criteria were inability to complete all trial procedures and follow-up visits, inability to keep the mouth open for at least 5 seconds continuously, inability to relax the jaw for 30 minutes, a sensitive gag reflex on physical examination, Friedman classification grade 4 tonsil size, insufficient exposure of the entire tonsil on physical examination, history of peritonsillar abscess, coagulation disorders (including the use of anticoagulants), contraindications for local or general anesthesia, evident tonsil asymmetry or other signs of possible malignant or premalignant neoplasm of the oropharynx, immunodeficiency, and pregnancy.

### Enrollment and Randomization

Patients were registered in an electronic data capturing system (Castor EDC^17^). Using computer generated random numbers, patients were randomly assigned to tonsillotomy or tonsillectomy treatment using stratified randomization, the strata being type of main tonsil concern. Stratification was used to control the potentially large influence of type of tonsil concern on study outcomes (strata: chronic tonsillitis, halitosis, tonsillolithiasis, dysphagia, and sleep apnea). Considering the different pathophysiological processes underlying the various concerns, small randomization imbalances could otherwise have a large influence on the analysis of observed treatment outcomes.

All patients randomly allocated to tonsillotomy were advised to start a gag-reflex desensitization training method. Patients were advised to slowly reduce their gag reflex by touching their tongue base and tonsils with a tooth brush each time they brushed their teeth. This method has been previously shown to reduce the gag reflex intensity in most patients within 2 weeks.^6^

### Interventions

#### CO_2_ Laser Tonsillotomy With Local Anesthesia

Carbon dioxide laser tonsillotomy was performed by trained surgeons (J.E.R.E.W.C., R.v.G., C.K., or H.M.B.) in ambulatory intervention rooms meeting the standard laser safety guidelines of the Dutch health council.^18^ A full operating room was available on-site for safety reasons. Each patient received acetaminophen, 1 g, orally prior to surgery. The patient was seated upright facing the surgeon and local anesthesia of the tonsil was achieved with xylocaine, 2%, containing adrenaline, 1:80 000 units, at a maximum of 5.4 mL. In patients with a substantial residual gag reflex, xylocaine, 10%, was sprayed on the peritonsillar area. After adequate anesthesia was accomplished, the CO_2_ laser was set between 25 and 30 W in continuous mode to distribute focused laser energy with a beam diameter of 3 mm. Patients were asked to breath in deeply; during slow exhalations, with the tongue depressed, the crypts of the tonsil were evaporated in a sweeping motion until full cryptolysis was accomplished. A smoke suction device was used to prevent smoke inhalation and to ensure the surgeon’s clear vision of the treatment area. In case of bleeding, coagulation was accomplished by pulling the laser out of focus. A step-by-step video protocol of this intervention has been published previously.^6^

All CO_2_ laser tonsillotomy procedures were performed in the leading clinical study center. The participating centers were close to the lead center (<2 hours driving time), enabling patients to travel for treatment.

#### Classic Dissection Tonsillectomy

Classic dissection tonsillectomy procedures were performed in all study centers. The patient was placed in a supine position, and general anesthesia with endotracheal intubation was induced. After applying a McIvor retractor, an Allis clamp was used to grasp the superior pole of the tonsil. Next, an incision was made on the anterior pillar of the tonsil to expose the tonsil. Using a tonsil clamp and scissors, the tonsil was removed. Hemostasis was ensured with gauze and gentle pressure for 5 minutes. If necessary, additional electrosurgery was performed for full hemostasis. After tonsillectomy, patients were admitted to the postanesthesia care unit and discharged the same day.

#### Postoperative Pain Medication

Postoperative analgesia for all patients consisted of acetaminophen, 500 mg, given as needed at a maximum of 4 times daily with 1000 mg each time. If acetaminophen was insufficient, diclofenac, 50 mg, was given as needed, with a maximum of 3 times daily for the first 3 days after surgery. If the combination acetaminophen and diclofenac was insufficient, tramadol was prescribed.

#### Patient Crossover

If deemed clinically necessary, patients could receive additional surgical treatments deviating from the assigned study group after their initial surgical treatment. Those additional tonsillotomy or tonsillectomy treatments were offered in line with the standard of care for symptoms to maintain a pragmatic and ethical randomized clinical trial design. No additional surgical procedures were performed within 6 weeks of the initial study assigned surgery. Patients who were randomized in the study but changed their mind and decided to not undergo their allocated treatment were asked for permission to continue to collect follow-up data on their tonsil symptoms and any surgical procedures they underwent.

### Clinical Data Collection

#### Preoperative Assessment

Before the surgical procedure, we collected demographic and clinical (risk) factors, including the preoperative tonsil-related symptoms and severity, medication use, and tobacco smoking status. Tonsil symptom severity and pain severity were collected both on ordinal (minimal, mild, moderate, and severe) and continuous visual analog scales (VAS) for consistency purposes. General health status was assessed using the 5-level EuroQol 5-Dimensions survey to measure health-related quality of life.^19^ To assess the influence of the tonsil-related symptoms on quantitative work productivity and activity impairment, we used the Work Productivity and Activity Impairment questionnaire.^20^

#### Surgical Complications and Early Postoperative Data Collection

During surgery, the surgeon graded the tonsil size using the Friedman grading scale. Duration of the intervention and any perioperative or postoperative complications were collected.

Data on perioperative and postoperative pain were collected using a 0-100 mm VAS and an ordinal scale (no pain or mild, moderate, or severe pain). Two weeks after surgery, patients reported when they felt fully recovered, when they returned to work, and the duration of analgesic use.

#### Long-term Follow-up Data Collection

Six months after the surgical procedure, data were collected on the presence of any tonsil-related symptoms, quality of life (5-level EuroQol 5-Dimensions survey), work productivity and activity impairment, and overall satisfaction (0-100 mm VAS). All patient-reported data were collected using digital questionnaires.

### Primary and Secondary Outcomes

The primary outcome was functional recovery time from surgery in days until patients reported being fully recovered, up to 2 weeks after surgery. We asked patients directly when they felt fully recovered from surgery. This primary outcome was selected because it is an important patient-centered outcome.^21^ Secondary outcomes included return to work within 2 weeks after surgery, postoperative pain scores, the duration of postoperative analgesic medication use, perioperative and postoperative complications, overall satisfaction, resolution of tonsil-related symptoms 6 months after surgery, and general health.

### Statistical Analysis

Baseline demographic and clinical characteristics in both groups are presented as means with SDs, medians with IQRs, or as numbers and percentages. Consistent with CONSORT clinical trial reporting guidelines, we did not perform statistical significance testing on the baseline characteristics of the randomized groups.^14^ The primary outcome of time to full recovery from surgery was analyzed only in patients who received a surgical intervention (modified intention-to-treat population, ie, by randomly assigned group but only among those with surgery). Functional recovery was not measurable among patients not undergoing surgery. Time was measured from the day of surgery. Time to recovery was graphically depicted in reverse Kaplan-Meier curves with pointwise confidence intervals and was compared using a log-rank test and hazard ratios using Cox regression.^22^ The proportional hazards assumption was tested using Schoenfeld residuals.^23^ Patients who did not reach full functional recovery within 2 weeks were censored at 2 weeks. Secondary outcomes of return to work within 2 weeks after surgery and duration of analgesic medication use were analyzed in a similar manner. The secondary outcome analyses on patient reported outcomes 6 months after surgery were performed on an intention-to-treat basis (randomized patients analyzed according to randomization). Characteristics 6 months after surgery were compared using χ^2^ tests, Fisher exact tests, Mann-Whitney tests, and unpaired *t* tests depending on the variable and its distribution. Within tonsillotomy and tonsillectomy group changes from baseline were assessed using Wilcoxon signed rank tests, paired *t* tests, and Fisher exact tests. Normality of data was assessed using the Shapiro-Wilk test. Two-sided *P* values were computed, and a significance level of .05 was used for all testing. Statistical analyses were performed using SPSS, version 27 (IBM) and JMP Pro, version 15 (SAS Institute Inc).

Data from a previous nonrandomized prospective study were used for sample size calculation.^24^ A 2-sided log-rank test with an overall sample size of 190 patients (95 in the tonsillectomy group and 95 in the tonsillotomy group) achieved 80.2% power at a .05 significance level to detect a tonsillotomy median functional recovery time of 8 days when the tonsillectomy group median survival time was 13.5 days within a 14-day total observation time.^25^

## Results

**Figure 1.**
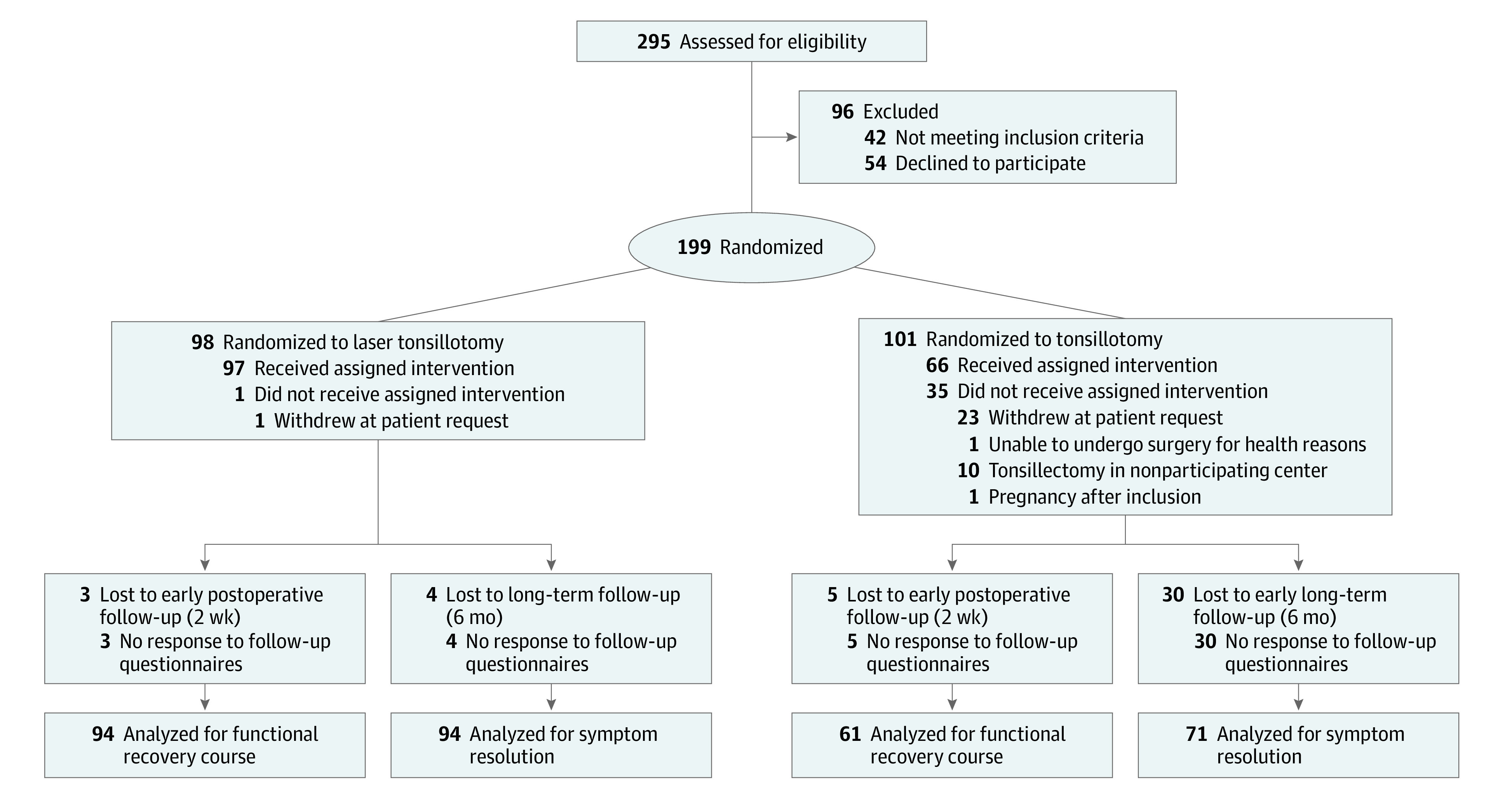
Patient Flow Diagram

A total of 199 patients were included and randomly allocated to tonsillotomy (98 patients) or tonsillectomy (101 patients). A treatment flowchart is presented in Figure 1.

Of 199 patients, 163 (82%) received their allocated treatment. In the tonsillotomy group, 1 patient withdrew from treatment after randomization. A total of 13 patients required a second tonsillotomy treatment within 6 months after the initial study treatment because of residual symptoms in 11 patients or unfinished primary tonsillotomy treatment for 2 patients. Eight patients who initially underwent tonsillotomy later received a tonsillectomy owing to recurrent symptoms. Three of these patients first received additional tonsillotomy. One patient had perioperative bleeding during tonsillotomy and received an elective tonsillectomy later for that reason. In the tonsillectomy group, 35 randomized patients did not receive the tonsillectomy within the study. In total, 23 patients requested withdrawal, 10 patients received tonsillectomy in a nonparticipating center, 1 patient developed back pain for which additional treatment was needed leading to cancellation of the tonsillectomy, and 1 patient became pregnant after randomization. One of the patients who withdrew later received a laser tonsillotomy in a hospital that did not participate in the study. There was no significant difference in baseline characteristics between patients receiving tonsillotomy or tonsillectomy who were treated and those who withdrew from treatment within the study, except for percentage of patients who were employed (127 of 163 [78%] vs 18 of 36 [50%], respectively; difference 95% CI, 5%-51%; *P* = .02).

A total of 94 patients in the tonsillotomy group and 61 patients in the tonsillectomy group were included in the modified intention-to-treat analyses of functional recovery after surgery, return to work after surgery, surgical complications, and early postoperative outcomes. A total of 94 patients in the tonsillotomy group and 71 patients in the tonsillectomy group were included in the long-term follow-up intention-to-treat analyses of symptom resolution (Figure 1).

### Baseline Characteristics

Demographic and clinical characteristics were similar between groups (Table 1). In both groups, most patients were female (tonsillotomy: of 98 patients, 69 [70%] were female and 29 [30%] were male; tonsillectomy: of 101 patients, 70 [69%] were female and 31 [31%] were male) and most patients reported moderately severe tonsil symptoms (tonsillotomy, 59 [61%]; tonsillectomy, 47 [62%]). The most common indications for surgery were recurrent infections with or without fever (130 of 199 reports [65%]) and tonsillolithiasis (64 of 199 reports [32%]).

**Table 1.** Baseline Demographic and Clinical Characteristics of Patients in the Tonsillotomy and Tonsillectomy Groups

Variable	No. (%) of patients	
	Tonsillotomy (n = 98)	Tonsillectomy (n = 101)
Demographic characteristic		
Sex		
Male	29 (30)	31 (31)
Female	69 (70)	70 (69)
Age, mean (SD), y	29 (10)	30 (8)
Tobacco smoking status		
Current	17 (18)	14 (18)
Former	24 (25)	16 (21)
Not smoking	56 (58)	46 (61)
Tonsil symptoms		
Chief tonsil concern		
Sore throat without fever	31 (32)	33 (33)
Sore throat with fever	33 (34)	33 (33)
Tonsillolithiasis	32 (33)	32 (32)
Snoring	2 (2)	2 (2)
Dysphagia	0	1 (1)
Self-reported severity of tonsil concerns (ordinal)		
Minimal	1 (1)	1 (1)
Mild	21 (22)	18 (24)
Moderate	59 (61)	47 (62)
Severe	16 (16)	10 (13)
Self-reported severity of tonsil concerns (continuous), mean (SD), mm^a^	57 (19)	59 (17)
QOL and work or activity impairment		
QOL (EQ-5D-5L) index score, median (IQR)^b^	0.87 (0.81-1.00)	0.87 (0.84-1.00)
EQ-5D-5L general health rating, median (IQR)^c^	80 (70-89)	80 (70-89)
Employed	70 (74)	57 (76)
WPAI overall work impairment, median (IQR), %^d^	7 (2-12)	5 (0-11)
WPAI interference with daily activities score, median (IQR)^e^	3 (2-6)	4 (2-6)

Abbreviations: EQ-5D-5L, 5-level EuroQol 5-Dimensions quality of life survey; QOL, quality of life; WPAI, Work Productivity and Activity Impairment questionnaire.

^a^ Measured using a 100-mm visual analog scale.

^b^ Range of the measurement instrument is −0.329 to 1.00.

^c^ Range of the measurement instrument is 0 to 100.

^d^ WPAI is evaluated only for patients who are employed.

^e^ Range of the measurement instrument is 0 to 10.

### Primary Outcome of Functional Recovery After Surgery

**Figure 2.**
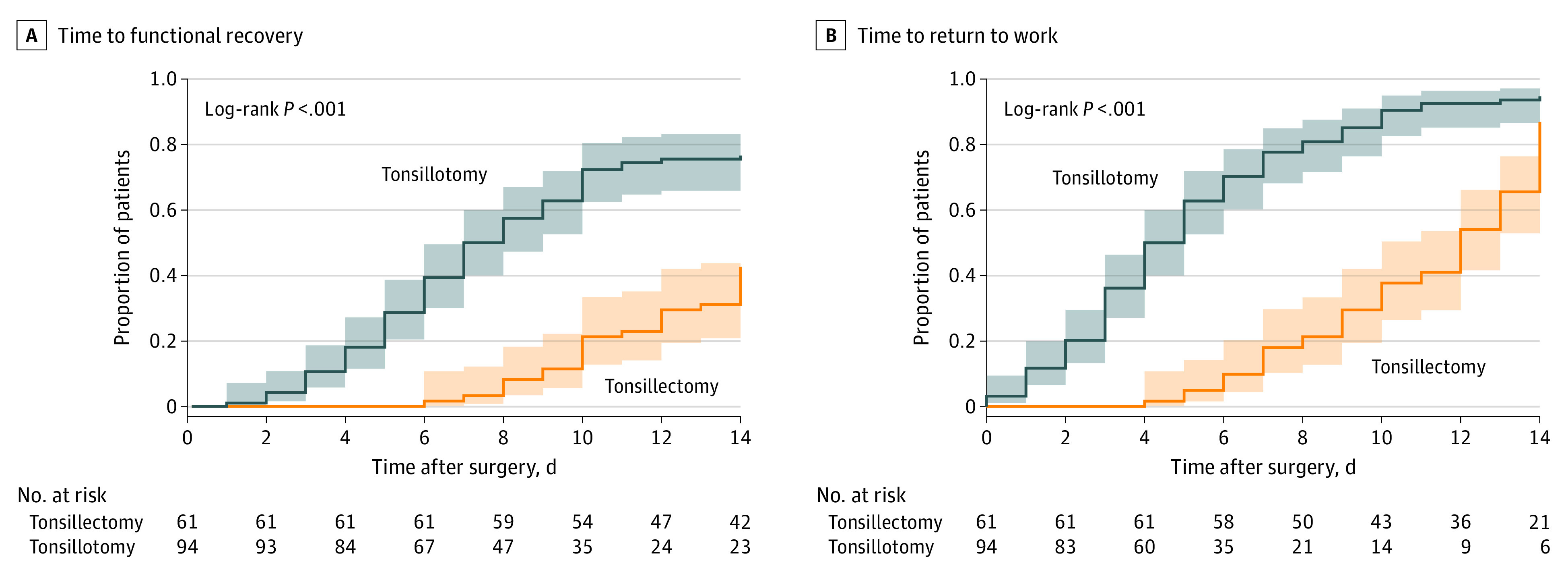
Reverse Kaplan-Meier Curves Showing the Proportion of Patients Functionally Recovered and Returned to Work Up to 2 Weeks After Tonsillectomy and Tonsillotomy Shaded areas indicate 95% CIs.

Two weeks after surgery, 72 (77%) patients in the tonsillotomy group were fully recovered compared with 26 (57%) patients in the tonsillectomy group. The time to recovery within 2 weeks after surgery was significantly different between the modified intention to treat tonsillotomy and tonsillectomy groups, with patients in the tonsillectomy group recovering substantially slower (hazard ratio for recovery after tonsillectomy vs tonsillotomy, 0.3; 95% CI, 0.2-0.5) Figure 2A. Median (IQR) full functional recovery time in the tonsillotomy group was 7.5 (5.0-12.0) days, and 22 patients were censored at 14 days for not reaching full recovery. In the tonsillectomy group, median recovery was not reached within 14 days, and 35 patients were censored at 14 days. At 12 days after surgery, the 25th percentile of full functional recovery was reached.

### Secondary Outcomes

#### Return to Work After Surgery

The time to return to work within 2 weeks was different between the tonsillotomy and tonsillectomy groups, with tonsillectomy patients returning to work later (hazard ratio for return to work for tonsillectomy vs tonsillotomy, 0.3; 95% CI, 0.2-0.4; *P* < *.*001) Figure 2B. Patients in the tonsillotomy group returned to work at a median (IQR) of 4.5 (3.0-7.0) days, whereas patients in the tonsillectomy group returned to work at a median (IQR) of 12.0 (9.0-14.0) days. At 14 days, 8 patients were censored in the tonsillectomy group and 5 patients were censored in the tonsillotomy group because they did not reach full recovery within 2 weeks.

#### Treatment and Surgical Complications

We terminated tonsillotomy treatment early in 3 of 97 patients (3%) because of increased bleeding of the tonsil shortly after initiating tonsillotomy treatment. For 1 of these 3 patients, bleeding was caused by active inflammation. After oral antibiotic treatment, a second tonsillotomy was successfully performed. The second patient crossed over to the tonsillectomy group, with tonsillectomy performed electively later, and the third patient experienced satisfactory symptom reduction after partial tonsillotomy treatment. We stopped 1 tonsillotomy treatment because of insufficient exposure of the tonsils (Mallampati scale class IV). No tonsillotomy treatments were stopped for patient discomfort or anxiety. Postoperative hemorrhage occurred in 2 of 97 patients (2%) in the tonsillotomy group and 8 of 66 patients (12%) in the tonsillectomy group (difference 95% CI, 2%-18%; *P* = *.*02). One of these patients experienced 2 separate postoperative hemorrhage events after tonsillectomy. The tonsillotomy hemorrhage events were controlled without intervention (n = 1) or with electrosurgery (n = 1). The tonsillectomy hemorrhage events were controlled without intervention (n = 2) or with electrosurgery (n = 6) performed under local anesthesia (n = 2) or general anesthesia (n = 4). After treatment, 2 of 97 (2%) patients in the tonsillotomy group and 1 of 66 patients (2%) in the tonsillectomy group developed wound infection. These infections were managed using oral antibiotics without hospital admission.

#### Early Postoperative Outcomes

##### Pain and Analgesic Medication Use

Perioperative pain was significantly lower in the tonsillotomy group compared with the tonsillectomy group (mean [SD] score, 36 [20] vs 58 [25] mm; effect size, 0.97; *P* < *.*001). Similarly, postoperative pain in the first 2 weeks was significantly lower in the tonsillotomy group (mean [SD] score, 42 [24] vs 66 [21] mm; effect size, 1.06; *P* < *.*001). More patients in the tonsillectomy group reported moderate (46% vs 30%; difference 95% CI, 1%-31%) and severe pain postoperative pain in the first 2 weeks (30% vs 10%; difference 95% CI, 8%-32%) compared with patients in the tonsillotomy group (both *P* < .001).

**Figure 3.**
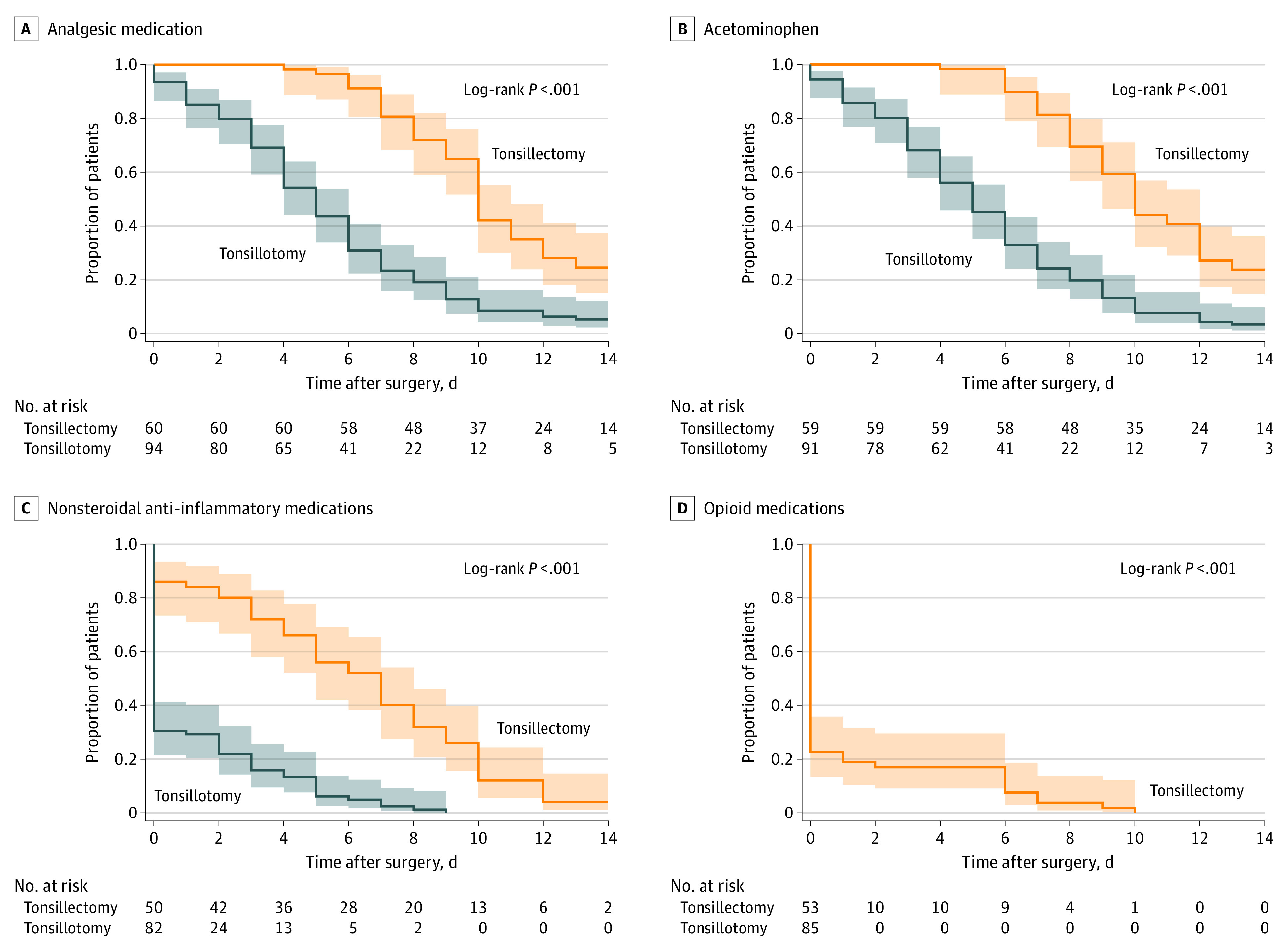
Use of Analgesic Medication During the First 2 Weeks After Tonsillotomy and Tonsillectomy Kaplan-Meier curves showing the proportion of patients using any type of analgesic medication (A) or a specific type of analgesic medication (B-D) during the first 2 weeks after surgery. Shaded areas indicate 95% CIs.

Analgesic medications used by patients in the tonsillotomy and tonsillectomy groups consisted of acetaminophen (94% vs 100%, respectively; difference 95% CI, 0% to −11%; *P* = *.*06), nonsteroidal anti-inflammatory drugs (39% vs 61%; difference 95% CI, −7% to −37%; *P* < *.*001), and opioid analgesics (1% vs 30%; difference 95% CI, −18% to −40%; *P* < *.*001). The survival distributions of days until no analgesic medication was required were significantly different between the tonsillotomy and tonsillectomy groups, with patients in the tonsillectomy group requiring analgesics longer (hazard ratio for analgesics no longer needed tonsillectomy vs tonsillotomy, 0.3; 95% CI, 0.2-0.4) (Figure 3A). The median (IQR) duration of analgesic medication use was 10 (8-13) days for the tonsillectomy group and 5 (3-7) days for the tonsillotomy group; 14 patients in the tonsillectomy group and 5 patients in the tonsillotomy group were censored for continued analgesic use at day 14. Survival distributions for individual drug classes acetaminophen (hazard ratio, 0.3; 95% CI, 0.2-0.4), nonsteroidal anti-inflammatory (hazard ratio, 0.2; 95% CI, 0.1-0.2), and opioids (hazard ratio, 0.1; 95% CI, 0.0 - 0.2) were also significantly different, with shorter use in the tonsillotomy group (all *P* < *.*001) Figure 3B-D. For the tonsillectomy group, 14, patients were censored at 14 days for the acetaminophen analysis, 2 patients for the nonsteroidal anti-inflammatory analysis, and 0 patients for the opioid use analysis. For the tonsillotomy group, 3 patients were censored at 14 days for the acetaminophen analysis and 0 patients for the nonsteroidal anti-inflammatory and opioid use analyses.

#### Long-term Follow-up

##### Resolution of Tonsil-Related Symptoms

Six months after randomization to tonsillotomy or tonsillectomy, the chief concern persisted in 54 of 94 patients (57%) after tonsillotomy and in 25 of 71 patients (35%) after tonsillectomy (difference 95% CI, 7%-37%; *P* = *.*005) (Table 2). For patients with remaining concerns, the severity of the chief concern decreased significantly both in the tonsillotomy (mean [SD] baseline, 57.6 [18.1] vs follow-up, 37.6 [22.1]; effect size 0.88; *P* = *.*01) and tonsillectomy (mean [SD] baseline, 54.4 [13.6] vs 23.9 [11.3] follow-up; effect size 2.24; *P* = *.*01) groups. Most patients with persistent symptoms in both the tonsillotomy (32 of 54; 59%) and tonsillectomy (16 of 25; 64%) groups reported mild symptoms 6 months after surgery. When measured on a continuous scale of 0 to 100, patients with persistent symptoms in the tonsillotomy group reported slightly higher symptom severity compared with the tonsillectomy group (mean [SD], 38 [22] vs 26 [13] mm; effect size 0.66; *P* = *.*02). For patients with remaining tonsil concerns, the distribution of the chief concern leading to surgery was similar to the baseline distribution among patients in the tonsillotomy group (baseline: sore throat without fever, 31 [32%]; sore throat with fever, 33 [34%]; tonsillolithiasis, 32 [33%]; snoring, 2 [2%]; and dysphagia 0 [0%] vs persistent symptoms: sore throat without fever, 16 [30%]; sore throat with fever, 14 [26%]; tonsillolithiasis, 23 [43%]; snoring, 1 [2%]; and dysphagia, 0 [0]%; *P* = *.*64) and in the tonsillectomy group (baseline: sore throat without fever, 33 [33%]; sore throat with fever, 33 [33%]; tonsillolithiasis, 32 [32%]; snoring, 2 [2%]; and dysphagia, 1 [1%] vs sore throat without fever, 9 [36%]; sore throat with fever, 8 [32%]; tonsillolithiasis, 6 [24%]; snoring, 2 [8%]; and dysphagia, 0 [0%]; *P* = *.*52), indicating that the type of tonsil concern did not influence treatment success.

**Table 2.** Characteristics of Patients With Persistent Symptoms After Tonsillotomy and Tonsillectomy as Well as Secondary Outcomes 6 Months After Surgery in All Patients Receiving Tonsillotomy or Tonsillectomy

Variable	No. (%) of patients		*P* value
	Tonsillotomy	Tonsillectomy	
**Tonsil symptoms in patients with persisting symptoms**			
Persistence of primary symptom that led to surgery^a^	54 (57)	25 (35)	.005
Self-reported severity of tonsil concerns in patients with persisting symptoms^b^			
Minimal	0	0	.46
Mild	32 (59)	16 (64)	
Moderate	13 (24)	8 (32)	
Severe	9 (17)	1 (4)	
Self-reported severity of tonsil concerns in patients with persistent symptoms (continuous), mean (SD), mm^c^^,^^d^	38 (22)	26 (13)	.02
Type of chief concern that led to surgery in patients with persistent symptoms^e^			
Sore throat without fever	16 (30)	9 (36)	.25
Sore throat with fever	14 (26)	8 (32)	
Tonsillolithiasis	23 (43)	6 (24)	
Snoring	1 (2)	2 (8)	
Dysphagia	0	0	
**Quality of life and work or activity impairment**			
No.	94	71	
QOL (EQ-5D-5L) index score, median (IQR)^b^^,^^f^	1 (0.85-1.00)	1 (0.87-1.00)	.20
EQ-5D-5L general health rating, median (IQR)^b^^,^^g^	80 (74-90)	85 (74-91)	.14
Employed^a^	73 (78)	54 (75)	.69
WPAI overall work impairment, median (IQR), %^b^	0 (0-10)	0 (0-0)	.001
WPAI interference with daily activities score, median (IQR)^b^^,^^h^	1 (0-3)	1 (0-2)	.24
**Patient satisfaction**			
Satisfaction with procedure score, median (IQR)^b^^,^^g^	77 (53-97)	87 (67-100)	.02

Abbreviations: EQ-5D-5L, 5-level EuroQol 5-Dimensions survey; QOL, quality of life; WPAI, Work Productivity and Activity Impairment questionnaire.

^a^ χ^2^ Test.

^b^ Mann-Whitney test.

^c^ Measured using a 100-mm visual analog scale.

^d^ Unpaired *t* test.

^e^ Fisher exact test.

^f^ Range of the measurement instrument is −0.329 to 1.00.

^g^ Range of the measurement instrument is 0 to 100.

^h^ Range of the measurement instrument is 0 to 10.

##### Quality of Life, Work Productivity, and Activity Impairment

At 6 months after surgery, patients in both the tonsillotomy (median [IQR] EQ-5D index, 1.00 [0.85-1.00]) and tonsillectomy (median [IQR] EQ-5D index, 1.00 [0.87-1.00]) groups reported excellent quality of life (*P* = *.*20). Patients in both the tonsillotomy (median [IQR] EQ-5D index: baseline, 0.87 [0.81-1.00) vs follow-up, 1.00 [0.85-1.00]; effect size, 0.24; *P* = *.*005) and tonsillectomy (median [IQR] EQ-5D index: baseline, 0.87 [0.84-1.00] vs follow-up, 1.00 [0.87-1.00]; effect size, 0.45; *P* = *.*003) groups experienced an increase in the quality of life index when compared with baseline. Patients in the both the tonsillotomy (median [IQR] WPAI work impairment at baseline, 7% [2%-12%] vs follow-up, 0% [0%-10%]; effect size, 0.15; *P* = *.*04) and tonsillectomy (median [IQR] WPAI work impairment at baseline, 5% [0%-11%] vs follow-up, 0% [0%-0%]; effect size, 0.39; *P* = *.*005) groups experienced improved participation in work and in daily activities compared with baseline (median [IQR] WPAI interference with daily activities at baseline, 3 [2-6] vs follow-up, 1 [0-3]; effect size, 0.65; *P* < *.*001; and for tonsillectomy at baseline, 4 [2-6] vs follow-up, 1 [0-2]; effect size, 1.19, *P* < *.*001).

##### Patient Satisfaction

At 6 months after surgery, overall patient satisfaction with treatment was slightly higher in the tonsillectomy group compared with the tonsillotomy group (median [IQR] score, 87 [67-100] vs 77 [53-97] mm; effect size, 0.35; *P* = *.*02). Similar percentages of patients in both the tonsillotomy and tonsillectomy groups would recommend the procedure to friends and family (80% vs 83%; difference 95% CI, −15% to 9%; *P* = *.*83).

## Discussion

Consistent with our primary hypothesis, this randomized clinical trial found that recovery, defined as time to both functional recovery and resumption of work, was shorter after tonsillotomy than after tonsillectomy. In addition, patients in the tonsillotomy group had less postoperative pain and shorter use of analgesic medication compared with patients in the tonsillectomy group. The types of analgesics used were also less potent. The tonsillectomy group had more postoperative hemorrhages. After 6 months, the chief concern persisted more often in patients randomized to tonsillotomy. We also found that 13% of patients in the tonsillotomy group required a second tonsillotomy treatment for remaining tonsil concerns. For patients in both the tonsillotomy and tonsillectomy groups who still experienced symptoms after 6 months, the severity of the symptoms decreased.

The shorter functional recovery, lower level of pain, and lower rate of hemorrhaging we found are consistent with previous studies. A prospective observational study by Lourijsen et al^24^ comparing laser tonsillotomy performed under local anesthesia to tonsillectomy found a shorter and less painful recovery period after tonsillotomy, lower mean pain 2 weeks after surgery (5.4 vs 7.7 on a 10 cm VAS), and less postoperative hemorrhaging (4.1% vs 6.5%). A randomized clinical trial by Ericcson et al^26^ comparing radiofrequency tonsillotomy performed under general anesthesia with tonsillectomy found a significantly faster resumption of normal activities with tonsillotomy (mean [SD], 6.4 [2.3] days) compared with tonsillectomy (10.6 [2.8] days); lower pain on postoperative days 1, 3, 5, and 10; lower analgesic medication requirements; and fewer postoperative hemorrhage events. Other studies have also found lower bleeding rates with tonsillotomy vs tonsillectomy.^27,28,29,30^

On the basis of direct surgical considerations, we believe that the differences in functional recovery, postoperative pain, and complications may be exclusively attributed to the less invasive nature of tonsillotomy. The postoperative wound after tonsillotomy may be comparable to a serious abrasion, whereas after tonsillectomy, tissue damage is more extensive, exposes the underlying constrictor muscle, and includes large blood vessels. This damage increases the risk of more serious postoperative hemorrhage. Similarly, after tonsillectomy, more and larger diameter sensory nerves are damaged, which adds to a significantly longer and more painful functional recovery period.

Eighteen patients who underwent tonsillotomy performed under local anesthesia required additional tonsillotomy or tonsillectomy treatment within 6 months of the initial study treatment because of residual symptoms or unfinished primary tonsillotomy treatment. All patients tolerated tonsillotomy treatment well; however, 3 tonsillotomy treatments were stopped because of increased bleeding. All initial tonsillectomy procedures were performed successfully under general anesthesia. At the 6-month follow-up, persistence of tonsil-related symptoms was significantly higher after tonsillotomy than after tonsillectomy, with 57% of patients in the tonsillotomy group still experiencing some level of symptoms compared with 35% of patients after tonsillectomy. However, patients with persistent tonsil-related concerns reported a significant decrease in symptom severity. Leaving some residual tonsil tissue is part of a successful tonsillotomy treatment. This is most likely the cause of the persistent tonsil-related concerns and may explain the difference found in this study. After the complete removal of the tonsils during tonsillectomy, tonsil symptoms persisting after surgery are unlikely. Throat concerns, however, can be caused by a variety of non–tonsil-related diseases, such as laryngopharyngeal reflux and pharyngitis. In fact, tonsillitis is often accompanied by pharyngitis.^31^ The coexistence of multiple anatomical disease generators may explain the persistence of patient-reported concerns after tonsillectomy.^32^ Consistent with the improvement in symptoms for patients with persistent symptoms in the tonsillotomy and tonsillectomy groups, both groups showed decreases in work impairment and of impairments in daily activities 6 months after surgery compared with baseline, with both groups reporting excellent quality of life at long-term follow-up. The percentage of patients who would recommend their treatment to others was similarly high for the tonsillectomy and tonsillotomy groups. This is surprising in light of the less effective symptom resolution that was provided by tonsillotomy. Perhaps the risk of needing a second tonsillotomy treatment for residual symptoms was offset by the benefits of local anesthesia, faster recovery, and overall lower complication rate.

### Limitations

This study has limitations. One important limitation is the uneven distribution of patients between the tonsillotomy and tonsillectomy groups who received their randomized treatment. We postulate that the higher patient withdrawal rate in the tonsillectomy group reflected the real-world hesitation to undergo tonsillectomy among patients. We emphasize that this withdrawal is not outcome dependent and therefore should not bias our results unless prognosis of the withdrawn patients differs from the general population. We compared the characteristics listed in Table 1 between patients who received treatment and patients who withdrew from the randomized surgical treatment and found no significant differences between groups, except for percentage of patients who were employed (78% vs 50%; *P* = *.*02). Thus, we have no indication that withdrawal biased our results. We also note that our modified intention-to-treat analysis of functional recovery and our intention-to-treat analysis of long-term follow-up symptom resolution represent real-world estimates of patient burden and treatment effect on symptoms.

We found no indication that the type of tonsil concern influenced treatment success. However, our study was likely underpowered to find any difference in specific subgroups. Further research should be conducted to assess potential differences.

The incidence of postoperative hemorrhage in the tonsillectomy group was higher than previously reported from retrospective studies.^32,33,34^ We believe that part of the higher postoperative hemorrhage rate in the present study may be attributed to our strict follow-up, which included questions directly related to complications, including postoperative hemorrhage. Other prospective studies have similar unexpected high rates of postoperative hemorrhages after cold steel dissection tonsillectomy.^35,36^

Finally, in the present study, patients with peritonsillar abscesses and patients with an indication for histopathologic analysis of the excised tonsil tissue (eg, to rule out malignant neoplasm) were excluded, and therefore the results of this trial do not apply to these populations. We recommend that patients with peritonsillar abscess be treated with tonsillectomy owing to the risk of recurrence of abscesses and the potentially lethal complications.^37^ Potential residual tonsil tissue after tonsillotomy in those cases is not desirable.^38^ Furthermore, when histopathologic analysis is required, laser tonsillotomy is not suitable because all of the tissue is evaporated by laser heating.

## Conclusions

This randomized clinical trial found that laser tonsillotomy performed under local anesthesia was a safe alternative to conventional tonsillectomy performed under general anesthesia for tonsil-related conditions among adults and was associated with a significantly shorter and less painful functional recovery period. Six-month follow-up data indicated that more tonsil concerns remained after tonsillotomy than after tonsillectomy, leading to a second tonsillotomy treatment for some patients. Depending on individual patient preferences, laser tonsillotomy performed under local anesthesia may be an alternative for conventional tonsillectomy performed under general anesthesia.

## Notes

Supplement 1.
Trial Protocol

Supplement 2.
Data Sharing Statement

## References

[1] Windfuhr JP, Toepfner N, Steffen G, Waldfahrer F, Berner R. Clinical practice guideline: tonsillitis II. surgical management. Eur Arch Otorhinolaryngol. 2016;273(4):989-1009. doi:10.1007/s00405-016-3904-x 26882912

[2] Hall MJ, Schwartzman A, Zhang J, Liu X. Ambulatory surgery data from hospitals and ambulatory surgery centers: United States, 2010. Natl Health Stat Report. 2017;(102):1-15. 28256998

[3] Senska G, Ellermann S, Ernst S, Lax H, Dost P. Recurrent tonsillitis in adults: quality of life after tonsillectomy. Dtsch Arztebl Int. 2010;107(36):622-628. doi:10.3238/arztebl.2010.0622 20948776 PMC2947847

[4] Seshamani M, Vogtmann E, Gatwood J, Gibson TB, Scanlon D. Prevalence of complications from adult tonsillectomy and impact on health care expenditures. Otolaryngol Head Neck Surg. 2014;150(4):574-581. doi:10.1177/0194599813519972 24691645

[5] Wong Chung JERE, van Benthem PPG, Blom HM. Tonsillotomy versus tonsillectomy in adults suffering from tonsil-related afflictions: a systematic review. Acta Otolaryngol. 2018;138(5):492-501. doi:10.1080/00016489.2017.1412500 29241412

[6] Wong Chung JERE, van Helmond N, van Geet R, van Benthem PPG, Blom HM. CO2-lasertonsillotomy under local anesthesia in adults. J Vis Exp. 2019;(153). doi:10.3791/59702 31762447

[7] Lister MT, Cunningham MJ, Benjamin B, . Microdebrider tonsillotomy vs electrosurgical tonsillectomy: a randomized, double-blind, paired control study of postoperative pain. Arch Otolaryngol Head Neck Surg. 2006;132(6):599-604. doi:10.1001/archotol.132.6.599 16785404

[8] Lee KD, Lee HS, Hong JC, . Diameter of vessels across the tonsillar capsule as an anatomical consideration for tonsillectomy. Clin Anat. 2008;21(1):33-37. doi:10.1002/ca.20562 18038453

[9] Koltai PJ, Solares CA, Koempel JA, . Intracapsular tonsillar reduction (partial tonsillectomy): reviving a historical procedure for obstructive sleep disordered breathing in children. Otolaryngol Head Neck Surg. 2003;129(5):532-538. doi:10.1016/S0194-5998(03)00727-7 14595276

[10] Younis RT, Lazar RH. History and current practice of tonsillectomy. Laryngoscope. 2002;112(8, pt 2)(suppl 100):3-5. doi:10.1097/00005537-200208001-00003 12172228

[11] Unkel C, Lehnerdt G, Schmitz KJ, Jahnke K. Laser-tonsillotomy for treatment of obstructive tonsillar hyperplasia in early childhood: a retrospective review. Int J Pediatr Otorhinolaryngol. 2005;69(12):1615-1620. doi:10.1016/j.ijporl.2005.08.017 16191441

[12] Ahmed J, Arya A. Lasers in tonsillectomy: revisited with systematic review. Ear Nose Throat J. 2021;100(1_suppl):14S-18S. doi:10.1177/0145561320961747 33048574

[13] Bredenkamp JK, Abemayor E, Wackym PA, Ward PH. Tonsillectomy under local anesthesia: a safe and effective alternative. Am J Otolaryngol. 1990;11(1):18-22. doi:10.1016/0196-0709(90)90165-R 2108585

[14] Moher D, Hopewell S, Schulz KF, . CONSORT 2010 explanation and elaboration: updated guidelines for reporting parallel group randomised trials. BMJ. 2010;340:c869. doi:10.1136/bmj.c869 20332511 PMC2844943

[15] World Medical Association. World Medical Association Declaration of Helsinki: ethical principles for medical research involving human subjects. JAMA. 2013;310(20):2191-2194. doi:10.1001/jama.2013.281053 24141714

[16] Federatie Medisch Specialisten. Richtlijn Ziekten van adenoïd en tonsillen (ZATT). Richtlijnendatabase.nl. Published 2014. Accessed January 4, 2022. https://richtlijnendatabase.nl/richtlijn/ziekten_van_adenoid_en_tonsillen_zatt/indicatie_voor_adenotomie_bij_zatt.html

[17] Castor. Castor EDC. 2022. Accessed January 4, 2022. https://www.castoredc.com/

[18] Koninklijke Nederlandse Academie Van Wetenschappen. Gezondheidsraad: commissie “laserveiligheid in de gezondheidszorg.” Published online 1992. Accessed August 9, 2020. https://pure.knaw.nl/portal/en/publications/gezondheidsraad-commissie-laserveiligheid-in-de-gezondheidszorg

[19] Herdman M, Gudex C, Lloyd A, . Development and preliminary testing of the new five-level version of EQ-5D (EQ-5D-5L). Qual Life Res. 2011;20(10):1727-1736. doi:10.1007/s11136-011-9903-x 21479777 PMC3220807

[20] Reilly MC, Zbrozek AS, Dukes EM. The validity and reproducibility of a work productivity and activity impairment instrument. Pharmacoeconomics. 1993;4(5):353-365. doi:10.2165/00019053-199304050-00006 10146874

[21] Hsu APP, Tan KL, Tan YB, Han HJ, Lu PK. Benefits and efficacy of tonsillectomy for recurrent tonsillitis in adults. Acta Otolaryngol. 2007;127(1):62-64. doi:10.1080/00016480500540501 17364331

[22] Altman D, Machin D, Bryant T, Gardner M. 2000: *Statistics with Confidence*. BMJ Books; 2001.

[23] Grambsch PM, Therneau TM. Proportional hazards tests and diagnostics based on weighted residuals. Biometrika. 1994;81(3):515-526. doi:10.1093/biomet/81.3.515

[24] Lourijsen ES, Wong Chung JERE, Koopman JP, Blom HM. Post-operative morbidity and 1-year outcomes in CO2-laser tonsillotomy versus dissection tonsillectomy. Acta Otolaryngol. 2016;136(10):983-990. doi:10.1080/00016489.2016.1183040 27224472

[25] Lakatos E. Sample sizes based on the log-rank statistic in complex clinical trials. Biometrics. 1988;44(1):229-241. doi:10.2307/2531910 3358991

[26] Ericsson E, Hultcrantz E. Tonsil surgery in youths: good results with a less invasive method. Laryngoscope. 2007;117(4):654-661. doi:10.1097/mlg.0b013e318030ca69 17415136

[27] Hessén Söderman AC, Ericsson E, Hemlin C, . Reduced risk of primary postoperative hemorrhage after tonsil surgery in Sweden: results from the National Tonsil Surgery Register in Sweden covering more than 10 years and 54,696 operations. Laryngoscope. 2011;121(11):2322-2326. doi:10.1002/lary.22179 21994191

[28] Windfuhr JP, Savva K. Aktuelle Studienlage zur Tonsillotomie: an update on tonsillotomy studies [in German]. HNO. 2017;65(1):30-40. doi:10.1007/s00106-016-0237-4 27670422

[29] Nemati S, Banan R, Kousha A. Bipolar radiofrequency tonsillotomy compared with traditional cold dissection tonsillectomy in adults with recurrent tonsillitis. Otolaryngol Head Neck Surg. 2010;143(1):42-47. doi:10.1016/j.otohns.2010.03.031 20620618

[30] Bender B, Blassnigg EC, Bechthold J, . Microdebrider-assisted intracapsular tonsillectomy in adults with chronic or recurrent tonsillitis. Laryngoscope. 2015;125(10):2284-2290. doi:10.1002/lary.25265 25876886

[31] Lechien JR, Akst LM, Hamdan AL, . Evaluation and management of laryngopharyngeal reflux disease: state of the art review. Otolaryngol Head Neck Surg. 2019;160(5):762-782. doi:10.1177/0194599819827488 30744489

[32] Bhattacharyya N, Kepnes LJ. Revisits and postoperative hemorrhage after adult tonsillectomy. *Laryngoscope*. 2014;124(7):1554-1556. 10.1002/lary.24541 24281921

[33] Arnoldner C, Grasl MCh, Thurnher D, . Surgical revision of hemorrhage in 8388 patients after cold-steel adenotonsillectomies. Wien Klin Wochenschr. 2008;120(11-12):336-342. doi:10.1007/s00508-008-0982-9 18709521

[34] Lowe D, van der Meulen J; National Prospective Tonsillectomy Audit. Tonsillectomy technique as a risk factor for postoperative haemorrhage. Lancet. 2004;364(9435):697-702. doi:10.1016/S0140-6736(04)16896-7 15325834

[35] Salonen A, Kokki H, Nuutinen J. Recovery after tonsillectomy in adults: a three-week follow-up study. Laryngoscope. 2002;112(1):94-98. doi:10.1097/00005537-200201000-00017 11802045

[36] Alexander RJ, Kukreja R, Ford GR. Secondary post-tonsillectomy haemorrhage and informed consent. J Laryngol Otol. 2004;118(12):937-940. doi:10.1258/0022215042790619 15667679

[37] Chang BA, Thamboo A, Burton MJ, Diamond C, Nunez DA. Needle aspiration versus incision and drainage for the treatment of peritonsillar abscess. Cochrane Database Syst Rev. 2016;12(12):CD006287. doi:10.1002/14651858.CD006287.pub4 28009937 PMC6463807

[38] Farmer SE, Khatwa MA, Zeitoun HM. Peritonsillar abscess after tonsillectomy: a review of the literature. Ann R Coll Surg Engl. 2011;93(5):353-355. doi:10.1308/003588411X579793 21943456 PMC3365450

