# Error in a Figure

**DOI:** 10.1001/jamanetworkopen.2022.5836

**Published:** 2022-03-16

In the Original Investigation titled “Time to Functional Recovery After Laser Tonsillotomy Performed Under Local Anesthesia vs Conventional Tonsillectomy With General Anesthesia Among Adults: A Randomized Clinical Trial,”^1^ published February 21, 2022, there was a typographic error in Figure 1. The amended phrase now reads “101 Randomized to tonsillectomy.” This article has been corrected.^1^
