# Supplementary material for: Time to Functional Recovery After Laser Tonsillotomy Performed Under Local Anesthesia vs Conventional Tonsillectomy With General Anesthesia Among Adults: A Randomized Clinical Trial
Source: JAMA Netw Open. 2022 Feb 21;5(2):e2148655. doi: 10.1001/jamanetworkopen.2021.48655 (PMC8861850; doi:10.1001/jamanetworkopen.2021.48655)
Supplement: Supplement 1. — Trial Protocol [file jamanetwopen-e2148655-s001.pdf]

C

RESEARCH PROTOCOL

THIS FILE HAS BEEN TRANSLATED ON April 28 2021 TO ENGLISH FOR REVIEWING PURPOSES.
THE ORIGINA FILE IS IN DUTCH

‘TOMTOM-study’- protocol

CO2-Lasertonsillotomy versus tonsillectomy in adults;
a randomized multicentre study

Keel-, neus- en oorheelkunde HagaZiekenhuis, ’s Gravenhage

J.E.R.E. Wong Chung MD, H. M. Blom MD PhD

Protocol ID

Title: CO2 laser tonsillotomy versus classic tonsillectomy in adults; a randomized multicentre study

Protocol ID	'TOMTOM-study' protocol
Version	5
Date	December 2017
Coordinating investigator	Drs. J.E.R.E. Wong Chung, ANIOS KNO, Haga ziekenhuis, Sportlaan 600, Den Haag tel. 0031-7022106561
Principal investigator	Dr. H.M. Blom, KNO-arts, HagaZiekenhuis, Sportlaan 600, Den Haag tel. 0031-7022106561
Initiator / Sponsor	Maatschap KNO, Haga ziekenhuis / MC Haaglanden , Den Haag, tel. 0031-7022106561.<br><br>Aanspreekpunt Haga ziekenhuis Dr. H.M. Blom, KNO-arts tel. 0031-7022106561
Independent expers(s)	Dr LJ Vleming
Laboratory	n/a

Table of contents

C RESEARCH PROTOCOL 1

1. Summary 6
2. Introduction 7
3. Aim of the study 8
   3.1. Study outcomes 8
   3.1.2. Secondary outcome measures 8
4. Study design 9
   4.1. Studie-design 9
5. Study population 11
   5.1. Population 11
   5.2. Inclusion criteria 11
   5.3. Exclusion criteria 11
6. Treatment specifications (intervention) 11
   6.1. Treatment to be investigated 11
   6.2. Control treatment 12
7. Methods 13
   7.1. Primairy endpoint 13
   7.2. Secundairy endpoints 13
   7.3. Randomisation 13
   7.4. Sample size calculation 13
   7.5. Study procedure 13
   7.6. Leaving the study prematurely 14
   7.7. Intervention prematurely / prematurely 14
   7.8. Section 10 WMO 15
   7.9. Adverse events (AE) and serious adverse event (SAE) 15
8. Statistical analysis 17
   8.1. Descriptive statistics 17
   8.2. Analysis 18
   8.3. (Statistical) Software 18
9. Ethical aspects 19
   9.1. Regulation statement 19
   9.2. Information provision and informed consent 19
   9.3. Patient insurance 19
   9.4. Risk Classification 19

10. Administrative aspects and publication 19
10.1. Handling and storage of data and documents 19
10.2. Amendments 19
10.3. Annual progress report 20
10.4. End of study report 20
11. STRUCTURED RISK ANALYSIS 20
12. Appendices 21
13. References 22

LIST OF ABBREVIATIONS AND RELEVANT DEFINITIONS

**CO_2**	Carbon dioxide
**KNO**	Keel, neus, oor
**MREC**	Medical Research Ethics Committee
**POK**	Poliklinische operatiekamer (Outpatient Surgery room)
**(S)AE**	(Serious) Adverse Event
**TE**	Tonsillectomy
**TO**	Tonsillotomy
**VAS**	Visual analogue scale
**WMO**	Wet Medisch-wetenschappelijk onderzoek met mensen

1. Summary

Background: In adults, a tonsillectomy can be performed for tonsil-related complaints according to the classic dissection technique under anesthesia. This procedure is associated with considerable morbidity. Outpatient CO2 laser tonsillotomy under local anesthesia appears to be an interesting alternative for a selected group of adults.

Objective: To evaluate the added value of CO2 laser tonsillotomy for the treatment of tonsil-related complaints.

Study design: The study will be conducted as an open multicenter randomized controlled trial. The patient will draw lots for a classic tonsillectomy or a laser tonsillotomy after informed consent.

Study population: Patients older than 18 years with tonsil-related complaints for which surgical intervention is indicated.

Intervention: A group will undergo a classic tonsillectomy under anesthesia. The other group will undergo a CO2 laser tonsillotomy under local anesthesia.

Outcome measures:
The results will be based on the following outcome measures: number of days postoperative recovery to resumption of daily activities, presence or absence of preoperative tonsil-related complaints, postoperative pain scores and analgesia use, procedure-related complications and patient satisfaction.

Risks and complications: For the number of clinic visits, measurement moments and actions, see the flow chart in chapter 3.

Complications of laser treatment:
- About 10% have not gotten rid of the complaints and have so many complaints that they undergo a classic dissection tonsillectomy
- In total, about 20% of patients undergo more than 1 laser treatment
- wound infection (3.4%)
- Bleeding requiring intervention (<1%)
- Allergic reaction to the local anesthetic (<1%)
- Side effects of the anesthetic (xylocaine 2% / adrenaline 1: 80,000) as described in the package insert (<1%)

Complications of a classic dissection tonsillectomy
- Bleeding requiring intervention (+ - 1.4%)
- Wound infection (+ - 2%)
- Complications of anesthesia (<1%)

2. Introduction

In adults, a tonsillectomy is performed using the classic dissection technique under general anesthesia. This procedure is associated with considerable morbidity. Outpatient CO2 laser tonsillotomy under local anesthesia appears to be an interesting alternative for a selected group of adults. Previous studies on outpatient CO2 laser tonsillotomy show that this procedure is associated with considerably less postoperative morbidity [1-7]. The rationale behind this therapy is that the tonsil crypts are the source of infection [8-9]. In contrast to a classic tonsillectomy, with a laser tonsillotomy, the crypts with the lymphatic tissue in between are superficially vaporized (cryptolysis). The tonsil capsule and peritonsillar space remain unaffected. The latter may explain why there is less (post) bleeding [10, 11, 12] and less pain, while the intended total cryptolysis prevents reinfection [3-7]. Furthermore, there is no need for general anesthesia and hospitalization. In addition to a medical benefit, this can also yield benefits from a financial point of view. An important side note to the available results of previous research is that most of the results are based on small patient groups where the patient characteristics are often lacking [14]. Few prospective follow-up results are known. For this reason, we conducted a follow-up study with the aim of looking at the feasibility, effectiveness and complications of a CO2 laser tonsillotomy[15].
In a period of four and a half years, 425 patients with chronic recurrent tonsillitis, fetor ex ore, swallowing complaints or obstructive complaints due to tonsil hypertrophy underwent a CO2 laser tonsillotomy after informed consent. This showed that patients were able to resume their daily activities and work after an average of 4.2 days. Of the 204 patients who could be followed for a long time (mean 16.8 months, range 1-40) it turned out that 69.6% eventually became completely free of complaints, in 19.1% there was such a reduction in complaints that no further intervention was desired, 10.3% were not free of symptoms after treatment and underwent a classic tonsillectomy. In 2.6% there was minimal bleeding which could be treated conservatively. In 8.6% antibiotics were prescribed shortly after the procedure, but these figures do not fully correspond to reality. In the early years, the GP regularly prescribed antibiotics because of the white discoloration of the wound bed. After better information provision, this number has decreased significantly. In 2010, 117 patients were treated with the laser and antibiotics were prescribed for a wound infection in 4 patients (3.4%). Two patients had a severe vagal reaction to the local anesthetic, causing the procedure to be prematurely discontinued.
A comparison with the classic tonsillectomy was not made in this study. Because of the expected potential of the CO2 laser tonsillotomy, we feel the need to perform a thorough comparative study with the classic tonsillectomy. The aim of this study is to provide more insight into postoperative recovery, effectiveness, complications (both in the short and longer term) and patient satisfaction after CO2 laser tonsillotomy compared to a classic tonsillotomy.

3. Aim of the study

The aim of this study is to compare the effectiveness of CO_2 laser tonsillotomy in a selected group of patients with a classic tonsillectomy. It is hypothesized that the number of days postoperative recovery after the laser procedure differs for at least 3 days from the classic tonsillectomy, that the effectiveness of the CO_2 laser tonsillotomy does not differ by more than 30% from the classic tonsillectomy and there are significantly better secondary outcomes.

3.1. Study outcomes

3.1.1. Primary outcome measure

- Number of days of post-operative recovery

3.1.2. Secondary outcome measures

- Presence or absence of the tonsil related complaint
- Number of episodes of tonsillitis per year objectified by the general practitioner or ENT specialist
- Number of antibiotics courses for tonsillitis each year
- Average number of days per year of sick leave due to tonsil-related complaints
- Presence / absence of halitosis
- Presence / absence of detritus
- Pain complaints during / after the procedure
- Complications
- Patient satisfaction with regard to the procedure and reduction of complaints (using VAS)
- Time to return to work / school resumption
- Number of days of sick leave in the year after surgery (second follow-up study)
- Cost Effectiveness laser tonsillotomy vs classic tonsillectomy (second follow-up study)
- Budget Impact laser tonsillotomy vs classic tonsillectomy (second follow-up study)

4. Study design

4.1. Study-design

The study will be conducted as an unblinded multicentre randomized controlled trial. The patient will be randomized for a classic tonsillectomy or a laser tonsillotomy.

The patients will be included in the study after written informed consent.
The CO2 laser tonsillotomies will take place in a treatment room that meets the laser criteria in the outpatient intervention room of the Haga Hospital, location Sportlaan (The Netherlands). The classic tonsillectomies will be performed in the operating rooms of the participating center where the patient has been enrolled.
The number of visits by the patient to the hospital is shown in the enclosed Flow-Chart (figure 1).

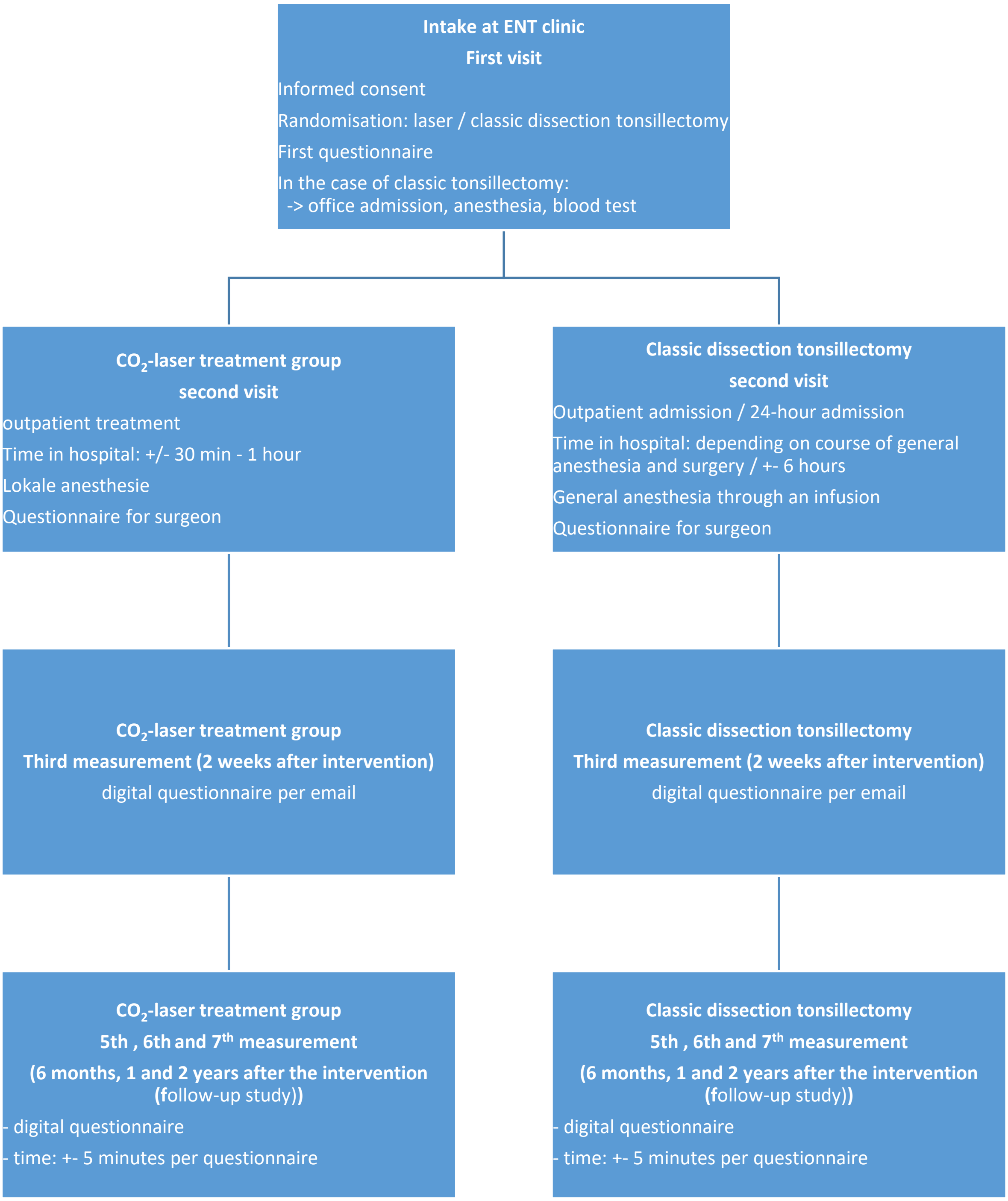
Intake at ENT clinic
First visit
Informed consent
Randomisation: laser / classic dissection tonsillectomy
First questionnaire
In the case of classic tonsillectomy:
-> office admission, anesthesia, blood test
CO_2-laser treatment group
second visit
outpatient treatment
Time in hospital: +/- 30 min - 1 hour
Lokale anesthesie
Questionnaire for surgeon
Classic dissection tonsillectomy
second visit
Outpatient admission / 24-hour admission
Time in hospital: depending on course of general anesthesia and surgery / +- 6 hours
General anesthesia through an infusion
Questionnaire for surgeon
CO_2-laser treatment group
Third measurement (2 weeks after intervention)
digital questionnaire per email
Classic dissection tonsillectomy
Third measurement (2 weeks after intervention)
digital questionnaire per email
CO_2-laser treatment group
5th , 6th and 7th measurement
(6 months, 1 and 2 years after the intervention (follow-up study))
- digital questionnaire
- time: +- 5 minutes per questionnaire
Classic dissection tonsillectomy
5th , 6th and 7th measurement
(6 months, 1 and 2 years after the intervention (follow-up study))
- digital questionnaire
- time: +- 5 minutes per questionnaire

5. Study population

5.1. Population

The study population will consist of men and women older than 18 years with tonsil-related complaints as described in more detail under indications.

5.2. Inclusion criteria

- Patients over 18 years old
- Indications:
  - Chronic / recurrent tonsillitis,> 4 tonsillitis / year (ZATT guideline, ENT association [17]) and / or
  - Halitosis and / or
  - Complaints of tonsillolithiasis and / or
  - Dysphagia and / or
  - OSAS (proven) caused by the tonsils

5.3. Exclusion criteria

- Troubled or uncooperative patient, at the discretion of the treating physician
- Inability to keep mouth open for long periods
  - [illegible] <5 seconds in a row
  - <30 minutes at intervals during which the jaw can be relaxed
- High strangulation reflex
- Tonsils that are too big (Friedman IV)
- Insufficient exposure of the entire tonsil
- History of peritonsillar abscess
- Hemorrhagic diathesis / use of blood thinning medication
- Allergy to local anesthetic
- Extensive history of co-morbidity in which anesthesia is not desirable or local anesthesia is not desirable
- Immune compromised patients

6. Treatment specifications (intervention)

6.1. Treatment to be investigated

The CO_2 laser tonsillotomies will be performed in the outpatient intervention room of the ENT department the Haga Hospital, location Sportlaan. The treatment room meets the criteria as formulated in the book “laserveiligheid in de gezondsheidszorg (“laser safety in health care”) (www.laserveiligheidindegezongdheidzorg.nl). Everyone present must wear safety glasses and a warning will be visible outside the room. 1000 mg of Paracetamol is given pre-operatively. The procedure is performed in a semi-reclining position. The tonsil and the superior, lateral and anterior sides of the pharyngeal arch are infiltrated with xylocaine 2% and adrenaline 1: 80,000. You will wait about 10 minutes for the anesthetic to take effect. It uses an F125 laser from Lumenis (Acupulse Surgitouch CO_2 laser system) with a beam diameter of 3 mm, with a power of up to 29 watts. The tonsil is presented with a tongue depressor after which the surface of the tonsils is evaporated layer by layer in a

sweeping motion. The patient is instructed to take a deep breath before the laser is activated and to exhale slowly during the actual laser treatment. The smoke created by the evaporation is continuously extracted by a smoke suction device.

Initially, the CO2 laser tonsillotomies will only be performed in the Haga Hospital. After training ENT doctors from other hospitals, it is examined whether the CO2 laser tonsillotomies can also take place in other clinics. If this turns out to be possible, an amendment will be submitted to request permission to expand.

6.2. Control treatment

The classic tonsillectomy is planned in one day admission or short clinical admission in the center of inclusion, this procedure is possible in all centers participating in this study. Peroperatively, a peripheral infusion is taken. The patient is placed in the supine position in the operating room and placed under general anesthesia, after which the patient is intubated. A mouth spreader is then placed in the mouth. An Alyss clamp is applied to the superior pole of the tonsil. An incision is then made through the anterior pillar of the tonsil to visualize the underlying capsule. The incision is made close to the anterior crease and is extended through the mucosa to the base of the tonsil. The tonsil is then removed with the help of a tonsil forceps. Meshes are applied to stop bleeding. After removing the gauze after 5 minutes, the wound bed is checked to see if it is dry and bipolar electro-coagulation is carried out if necessary. The mouth spreader is removed when the wound bed is dry. After the operation, the patient is admitted to the ward.

Post-operative pain relief both procedures:
Prescription paracetamol 500mg 4dd2, use if necessary at the patient's own discretion.
Prescription diclofenac 50mg 3dd1, use if necessary for the first 3 days at the patient's discretion.

7. Methods

7.1. Primairy endpoint

- Number of days post-operative recovery to resumption of daily activities

7.2. Secundairy endpoints

- In / or absence of the tonsil-related complaint
- Number of episodes of tonsillitis per year objectified by the general practitioner or ENT specialist
- Number of antibiotic courses for tonsillitis each year
- Average number of days per year of absenteeism due to the tonsil-related complaints
- Presence / absence of halitosis
- Presence / absence of detritus
- Pain complaints during / after the procedure
- Complications
- Patient satisfaction with regard to the procedure and reduction of complaints (using VAS)
- Time to return to work / school resumption
- Number of days of sick leave in the year after surgery (second follow-up study)
- Cost Effectiveness laser tonsollotomy vs classic tonsillectomy (second follow-up study)
- Budget Impact laser tonsollotomy vs classic tonsillectomy (second follow-up study)

7.3. Randomization

After informed consent, the patient will be randomized to one of the two treatments. It is not possible to perform the study blinded. The drawing will be done by means of a stratification randomization, taking into account the indication. About 50% of the patient group will be randomized to a classic tonsillectomy and about 50% to a laser tonsillotomy.

7.4. Sample size calculation

Using data from a previous non-randomized prospective study our group conducted a sample size calculation prior to conducting the present the randomized controlled trial.[21] . A two-sided log-rank test with an overall sample size of 190 subjects (95 in the TE group and 95 in the TO group) achieves 80.2% power at a 0.05 significance level to detect a TO median recovery time of 8 days when the TE group median survival time is 13.5 days within a 14-day total observation time.[22] .

7.5. Study procedure

To evaluate the primary and secondary outcome measures, the patient will be asked to complete a digital evaluation form preoperatively, 2 weeks, 6 months, 1 year and 2 years after the procedure (follow-up study). These forms will be offered by e-mail. In the event of recurrent complaints after the procedure, the patient is asked to go to the ENT doctor /

general practitioner for assessment and to report this to the investigator (by telephone / email). This will also be re-evaluated in the fixed measurement moments.

The operator will fill in a number of questions related to the procedure.

Laser procedure:

- Duration of the laser procedure
- Total duration of outpatient visit
- Amount of local anesthesia administered (number of cartridges)
- Pain during the procedure (VAS score) (patient perception)
- Use of supplemental anesthetic, and the amount thereof
- Coagulation required / not required during procedure
- Complications during surgery

Classic TE procedure:

- Duration procedure (cutting time)
- Duration of hospitalization
- Pain relief administered during surgery (medication, dosage)
- Pain related to procedure (injecting an IV, etc.) (VAS score) (perception pt)
- Coagulation required / not required during procedure
- Complications during surgery

7.6. Leaving the study prematurely

The patient can withdraw from the study at any time. The physician may withdraw the patient from the study at any time for medical reasons on the patient's behalf.

7.7. Intervention prematurely / prematurely

Both the physician and the patient can decide during the procedure to terminate the intervention for medical or other reasons.

Safety Considerations

7.8. Section 10 WMO

In accordance with section 10, subsection 4 of the Medical Scientific Research with Human Subjects Act (WMO), the sponsor will discontinue the study if there is a sufficiently substantiated suspicion that continuation of the study will endanger the health or safety of the patient. The sponsor will, without undue delay, inform the accredited MREC of the temporary discontinuation and the reason for stopping the study. The study will be discontinued pending a future positive decision from the accredited MREC. The researcher will ensure that all involved are informed.

7.9. Adverse events (AE) and serious adverse event (SAE)

With both treatments there is a risk of subsequent bleeding during the procedure or postoperatively. In the case of the classic TE, this is approximately 1.4% (2007) in the HAGA hospital where intervention is required. There is also a risk of infection after the treatment with both treatments, this risk is comparable for both interventions.
At present, there is no evidence that transmission of "transmissible spongiform encephalitis" can occur through inhalation of the smoke and / or vaporized tissue. In theory, however, there is a risk for this with CO2 laser tonsillotomy. Despite the fact that there are no specific guidelines for plumes of smoke in relation to variant Creutzfeld Jacob Disease and the risk of contamination of the treating physician and operating assistants is estimated to be very low, the safety guidelines regarding laser treatment recommend the use of an efficient filtering evacuation system. [14, 16]
Administration of local anesthesia may result in anaphylactic shock or a systemic reaction as described in the side effects of xylocaine / adrenaline.

In connection with these potentially unwanted consequences / side effects of the treatment, several precautions will be taken.

- An on-call ENT specialist is available at all times when a complication occurs that requires re-intervention / evaluation.
- Patient saturation and heart rate are monitored throughout the procedure.
- The procedures will take place in clinics where staff with BLS certificates will be present at all times. An anaphylactic set and respiratory equipment will also be available.
- The treatment room where the laser treatment takes place meets the criteria as formulated in the book “laser safety in health care” (www.laserveiligheidindegezongdheidzorg.nl).

If there is an (S)AE, this will be reported via the ToetsingOnline web portal to the accredited MREC that has approved the protocol. If there is an SAE, this will be reported to the accredited MREC within 15 days. Death or life-threatening condition will be reported to the Medical Ethics Committee within 7 days and initial reporting will be completed within 8 days.
An annual safety report with an overview of all SAEs will be sent to the MREC.

8.3 Monitoring

The research will be monitored by the Haga Hospital's monitor pool according to the Haga Hospital's monitoring plan.

8. Statistical analysis

8.1. Descriptive statistics

This study will contain different types of data. Both qualitative data and quantitative data are used. The measurements are made using four measuring scales: ordinal, nominal, interval and ratio. Which scale is used depends on the variable.

The following patient characteristics will be stored in the database which will be analyzed later:

- Date of birth
- Comorbidities
- Medication use
- Smoking / not smoking
- Head complaint (Sore throat with fever, sore throat without fever, dysphagia, detritus, halitosis, OSAS)
- Presence of other complaints (Sore throat with fever, sore throat without fever, dysphagia, detritus, halitosis, OSAS)
- Number of antibiotics used in the past year in connection with tonsil-related complaint
- Number of days of absenteeism from work / school related to tonsil-related complaints
- VAS score with regard to complaints (to be completed per complaint)
- Friedman classification (see appendix 1)

The variables that are analyzed related to the procedure are the following:
Laser procedure:

- Duration of the laser procedure
- Total duration of outpatient visit
- Amount of local anesthesia administered (number of cc)
- Pain during the procedure, VAS score (perception pt). Reduce pain score 30 minutes after surgery, 1 day after surgery, 2 weeks after surgery.
- Use of additional anesthetic (number of cc)
- Coagulation required / not required during procedure
- Complications during surgery

Classic TE procedure:

- Duration of hospitalization
- Pain relief administered during surgery (medication, dosage)
- Pain related to procedure, VAS score (patient perception). Pain score decrease 2 weeks after surgery.
- Complications during surgery

The variables to assess effectiveness and patient satisfaction include the following:

- Presence / absence of the complaints for which the intervention was undergone
- Duration of recovery period (time to return to work / resumption of daily activities) in days
- Duration of use of painkillers in number of days
- Satisfaction with the procedure (VAS score)
- Complaints after surgery (on / absent, VAS score)
- Short and long term complications (bleeding, infection, abscess)
- Number of laser sessions required
- If patient eventually underwent classic tonsillectomy
- Number of antibiotics used in the period after surgery in connection with tonsil-related complaint
- Number of days of absenteeism from work / school related to tonsil-related complaints after the procedure
- The patient would / would not recommend the procedure to others

8.2. Analysis

All analyzes will be performed with an intention-to-treat analysis.
A two sided p value <0.05 will be considered significant for all analyses. Survival analysis will be used to compare the primary outcome of recovery time. Survival analysis will also be used to compare time to return to work and postoperative analgesic use. Secondary outcome measures on a continuous scale will be compared using unpaired T-tests or Mann-Whitney U tests, depending on the normality of data (tested using Shapiro-Wilk test). Proportions will be compared using Fisher exact tests.

8.3. (Statistical) Software

The data will be stored in the databases of the CastorEDC system. The static analysis will be performed using the SPSS software package for Windows, version 24.0.

9. Ethical aspects

9.1. Regulation statement

This study will take into account the principles related to medical ethics as described by the World Medical Association in the 'WMA Declaration of Helsinki - Ethical Principles for Medical Research Involving Human Subjects (2013)' [19] and as described in the Medical Scientific Research with Humans Act (WMO).

9.2. Information provision and informed consent

The patients who are eligible to participate in the study will be informed by the doctor about the advantages and disadvantages of the different interventions. The possibility of conservative medical treatment versus surgery will also be discussed. After providing both oral and written information, the patient is given the opportunity to choose whether to participate in the study. The patient can indicate the time needed to decide whether or not to participate in the study. The maximum reflection period is 2 weeks. Additional information can be found by the patient via our informative website.

9.3. Patient insurance

All subjects participating in this study in all participating centers are covered by the WMO subject insurance that has been taken out by the Haga Hospital with Centramed.

9.4. Risk Classification

Moderate risk.

10. Administrative aspects and publication

10.1. Handling and storage of data and documents

The data obtained during this study will be treated confidentially and anonymously. The personal data will be treated in accordance with the Personal Data Protection Act (Wbp). The data will be encrypted where possible and a personal identification code list will be generated. The key to this code will be guarded by the principal investigator. Only the principal investigator and coordinating investigator have access to the source data. Data is kept for a period of ten years.

10.2. Amendments

All substantial changes will be communicated to the MREC and must be approved by them before these changes can be put into practice.
A substantial change is defined as a change from the application approved by the MREC that affects:

- the safety of the physical or mental integrity of the participants;
- the scientific value of the study;
- the management of the study;
- the quality or safety of the intervention used in the study

Substantial changes will not be communicated to the MREC.

10.3. Annual progress report

Once a year, the researcher will send a summary of the progress of the process to the accredited MREC. Information about the number of participants, number of participants who completed the study, (S) AEs, changes and problems are communicated.

10.4. End of study report

The researcher informs the accredited MREC within 8 weeks after the end of the study. The end of the study is defined as the last visit of the last study participant. In the event that the study is prematurely terminated, the investigator will inform the MREC of this, including the reasons for this.
Within one year of the end of the study, the investigator will submit a final report with the results of the study. It is expected that at least 2 publications in international journals will follow as a result of this research. These will be sent to the MREC.

11. STRUCTURED RISK ANALYSIS

N/A

12. Appendices

Figure 2 :. Tonsil size, Friedman classification.

0. *Status after tonsillectomy*
1. *Tonsils hidden between the pharyngeal arches*
2. *Tonsils extend just outside the pharyngeal arches*
3. *Tonsils enlarged beyond the pharyngeal arches, but not over the median line*
4. *tonsils enlarged to (over) the median line, "Kissing Tonsils"*

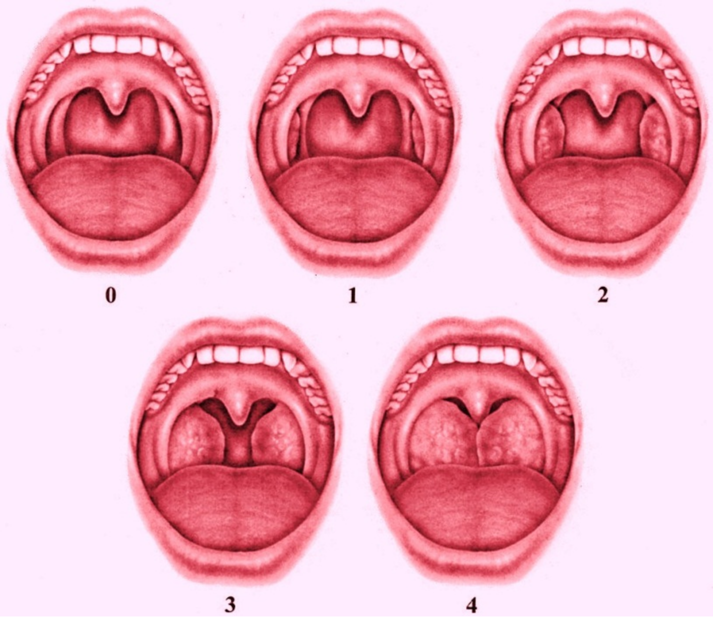

13. References

1. Lowe D,van der Meulen J Cromwell D, et al, Key messages from the National Prospective Tonsillectomy audit. Laryngoscope 2007; 117:717
2. Hoddeson EK, Gourin CG: Adult tonsillectomy: current indications and outcomes. Otolaryngology-- head and neck surgery : official journal of American Academy of Otolaryngology-Head and Neck Surgery 2009,
3. Datema FR, Holland CTQ, Blom HM. KTP/532 laser-tonsillectomy and CO_2 laser-tonsillotomy. Ned. Tijdsch. KNO-heelkd. 2007; 13: 65-70. Article in Dutch.
4. Krespi YP, Ling E. Laser-assisted serial tonsillectomy. J. Otolaryngol. 1994; 23: 325-327.
5. Andrews PJ, Latif A. Outpatiënt laser tonsillar ablation under local anaesthetic. Eur. Arch. Otorhinolaryngol. 2004; 261:551-554.
6. Remacle M, Keghian J, Lawson G, Jamart J. Carbon-dioxide laser-assisted tonsil ablation for adults with chronic tonsillitis: a 6 month follow-up study. Eur Arch Otorhinolaryngol. 2003; 260: 456-459.
7. Finkelstein Y, Talmi YP, Ophir D, Berger G. Laser cryptolysis for the treatment of halitosis. Otolaryngol Head Neck Surg. 2004 Oct;131(4):372-7
8. Perry M, Whyte A. Immunology of the tonsils. Immunol Today. 1998; 19(9): 414-421.
9. Rio AC, Franchi-Teixeira AR, Nicola EM. Relationship between the presence of tonsilloliths and halitosis in patients with chronic caseous tonsillitis. Br Dent J. 2008; 26; 204(2):E4.
10. Acevedo JL, Shah RK, Brietzke SE: Systematic review of complications of tonsillotomy versus tonsillectomy. Otolaryngology head and neck surgery: official journal of American Academy of Otolaryngology-Head and Neck Surgery 2012, 146(6):871-879.
11. Hessen Soderman AC, Ericsson E, Hemlin C, Hultcrantz E, Mansson I, Roos K, Stalfors J: Reduced risk of primary postoperative hemorrhage after tonsil surgery in Sweden: results from the National Tonsil Surgery Register in Sweden covering more than 10 years and 54,696 operations. *The Laryngoscope* 2011, 121(11):2322-2326.
12. Reichel O, Mayr D, Winterhoff J, de la Chaux R, Hagedorn H, Berghaus A: Tonsillotomy or tonsillectomy?--a prospective study comparing histological and immunological findings in recurrent tonsillitis and tonsillar hyperplasia. *European archives of oto-rhino-laryngology : official journal of the European Federation of Oto-Rhino-Laryngological Societies* 2007, 264(3):277-284.
13. Windfuhr JP, Savva K, Dahm JD, Werner JA: Tonsillotomy: facts and fiction. *European archives of oto-rhino-laryngology : official journal of the European Federation of Oto-Rhino-Laryngological Societies* 2015, 272(4):949-969.
14. British Association of Otorhinolaryngologist and Head en Neck surgeons (2001) Single use instruments for tonsil and adenoid surgery, alterative techniques. Department of Health
15. Windfuhr JP, Werner JA: Tonsillotomy: it's time to clarify the facts. European archives of oto-rhino-laryngology : official journal of the European Federation of Oto-Rhino-Laryngological Societies 2013, 270(12):2985-2996.
16. Department of Health. Minimise transmission risk of CJD and vCJD in healthcare settings (publised 2012, last updated 10-2015). https://www.gov.uk/government/publications/guidance-from-the-acdp-tse-risk-management-subgroup-formerly-tse-working-group
17. Richtlijn Ziekten van Adenoïd en Tonsillen, www.cbo.nl

18. Stafford N, von Haacke N, Sene A, Croft C. The treatment of recurrent tonsillitis in adults. J Laryngol Otol 1986;100:175-7.
19. http://www.wma.net/en/30publications/10policies/b3/
