# Supplementary material for: Time to Functional Recovery After Laser Tonsillotomy Performed Under Local Anesthesia vs Conventional Tonsillectomy With General Anesthesia Among Adults: A Randomized Clinical Trial
Source: JAMA Netw Open. 2022 Feb 21;5(2):e2148655. doi: 10.1001/jamanetworkopen.2021.48655 (PMC8861850; doi:10.1001/jamanetworkopen.2021.48655)
Supplement: Supplement 2. — Data Sharing Statement [file jamanetwopen-e2148655-s002.pdf]

Data Sharing Statement

Wong Chung. Time to Functional Recovery After Laser Tonsillotomy Performed Under Local Anesthesia vs Conventional Tonsillectomy With General Anesthesia Among Adults. *JAMA Netw Open*. Published February 21, 2022. doi:10.1001/jamanetworkopen.2021.48655

Data
Data available: Yes
Data types: Deidentified participant data
How to access data: All data collected in this study will be made available in a timely fashion to the scientific community for use in joint analyses on reasonable request after approval of the local Research Ethics Committee and with a signed data access agreement. All patient data is stored in an electronic data capture system
When available: With publication

Supporting Documents
Document types: None

Additional Information
Who can access the data: All data collected in this study will be made available in a timely fashion to the scientific community for use in joint analyses on reasonable request after approval of the local Research Ethics Committee and with a signed data access agreement. All patient data is stored in an electronic data capture system
Types of analyses: Meta analysis upon reasonable request
Mechanisms of data availability: All data collected in this study will be made available in a timely fashion to the scientific community for use in joint analyses on reasonable request after approval of the local Research Ethics Committee and with a signed data access agreement. All patient data is stored in an electronic data capture system
